# Care and primary health care access indicators among Brazilian adults with diabetes mellitus, National Health Survey 2019

**DOI:** 10.1590/1980-549720260005

**Published:** 2026-03-16

**Authors:** Luís Antônio Batista Tonaco, Bárbara Aguiar Carrato, Filipe Malta dos Santos, Regina Tomie Ivata Bernal, Deborah Carvalho Malta

**Affiliations:** I Universidade Federal de Minas Gerais, School of Nursing - Belo Horizonte (MG), Brazil.; II Fundação Educacional Lucas Machado - Belo Horizonte (MG), Brazil.

**Keywords:** Diabetes mellitus, Health status indicators, Primary health care, Health surveys, Brazil

## Abstract

**Objective:**

To analyze indicators of access to health services and care among adults with Diabetes mellitus in Brazil, according to Primary Health Care coverage.

**Methods:**

A cross-sectional study using data from the 2019 National Health Survey (PNS). Only individuals who self-reported a diagnosis of Diabetes mellitus were selected. Explanatory variables represented indicators of care and access among adults with a Diabetes mellitus diagnosis. Prevalence and 95% confidence intervals (95%CI) were calculated for the indicators. The association with registration in a Family Health Unit was verified by bivariate analysis. Indicators with p≤0.05 were included in a logistic regression model and, subsequently, in a model adjusted for sex and age.

**Results:**

Among the Brazilian adult population, 8.2% (95%CI 7.9-8.6) self-reported a diagnosis of Diabetes mellitus. After adjustment, individuals with diabetes living in households registered in a Family Health Unit were more likely to have had their last consultation in a Primary Health Care Unit (OR=2.13; 95%CI 1.71-2.64), to have been seen by a specialist physician after referral (OR=1.97; 95%CI 1.49-2.60), and to report severe limitations in usual activities due to the condition (OR=1.65; 95%CI 1.05-2.62). On the other hand, they were less likely to have been seen by the same doctor as in previous consultations (OR=0.65; 95%CI 0.53-0.81) and to have undergone an eye exam in the past year (OR=0.73; 95%CI 0.58-0.90).

**Conclusion:**

Disparities exist in care and access to Primary Health Care services among Brazilian adults with diabetes.

## INTRODUCTION

Diabetes mellitus (DM) is a serious chronic disease characterized by elevated blood glucose levels, resulting from abnormalities in β-cell function that affect insulin action^1^. The condition is classified into several types, with type 1 and type 2 being the most prevalent^2^. DM is associated with multiple complications, including retinopathy, nephropathy, diabetic foot, amputations, and an increased risk of cardiovascular disease and stroke^2^ ^,^ ^3^. When poorly controlled, DM contributes to adverse clinical outcomes, such as frequent hospitalizations, functional impairments, and increased mortality^3^.

The condition affects approximately 3% of the global population and is projected to increase through 2030^3^ ^,^ ^4^. Diabetes ranks 9^th^ among the leading causes of Disability-Adjusted Life Years (DALYs) lost worldwide^4^. In Brazil, DM also constitutes a significant public health concern^3^ ^,^ ^4^. Data from the 2013 National Health Survey (*Pesquisa Nacional de Saúde* - PNS) indicated a self-reported prevalence of 6.2%^3^, which increased to 8% by 2019, with markedly higher rates observed among individuals of lower socioeconomic status^3^.

In 2014, the Ministry of Health issued a decree establishing guidelines for structuring care pathways for individuals with noncommunicable chronic diseases (NCDs), including diabetes mellitus^5^. This initiative aims to enhance healthcare for people with NCDs through continuous and high-quality monitoring^5^ ^,^ ^6^. The primary objective is to prevent complications, thereby reducing hospitalizations and avoidable deaths^5^ ^,^ ^6^.

Access to health services is a fundamental factor in positively influencing the course of diseases and promoting health^7^. Generally, this concept encompasses the timely and appropriate utilization of available services to achieve optimal health outcomes^7^. In Brazil, access to health services is facilitated through programs such as the Family Health Strategy (*Estratégia Saúde da Família* - ESF), which expand the coverage of Primary Care (PC) nationwide, supporting prevention, early diagnosis, and continuous follow-up^8^ ^,^ ^9^.

In the context of diabetes, PC must ensure not only timely access but also continuous clinical follow-up, regular examinations, and educational interventions focused on self-care^10^. Monitoring indicators related to disease management and healthcare provision is essential for evaluating the quality of care delivered to individuals with DM, as it allows the identification of inequalities in access and service delivery, providing a foundation for the development and improvement of public policies.

The PNS serves as an important instrument for evaluating such indicators, as its questionnaire includes items on self-reported diagnosis of DM and the care received by affected individuals^6^. Accordingly, this study aimed to analyze indicators of access to health services and care among adults with DM in Brazil, according to primary care coverage.

## METHODS

### Study design and data source

This cross-sectional study utilized data from the 2019 PNS, conducted by the Ministry of Health in collaboration with the Brazilian Institute of Geography and Statistics (*Instituto Brasileiro de Geografia e Estatística -* IBGE). The PNS data are publicly available through the survey’s official repository^11^.

### Background

The PNS is a nationwide household survey, with a questionnaire structured in three sections: the first addresses household characteristics; the second is directed to all residents; and the third is applied to a single selected resident aged 15 years or older^12^. The survey allows for estimates to be obtained for urban and rural areas, as well as for the country’s macro-regions, federative units, capitals, and metropolitan regions^12^.

### Participants

The final sample of the 2019 PNS comprised 94,114 households with completed interviews, yielding a response rate of 93.6%^11^ ^,^ ^12^. The target population included individuals aged 15 years or older residing in permanent private dwellings, defined as buildings intended exclusively for housing^12^. Households located in census sectors with special characteristics or low population density, such as indigenous communities, military institutions (barracks and bases), and temporary accommodations, were excluded from the sample^12^. Detailed information on the survey methodology is available in specific publications^11^ ^,^ ^12^. For the present study, only individuals aged 18 years or older who responded affirmatively to the question: “*Has a doctor ever diagnosed you with diabetes?*” were included, resulting in a study population of 7,374 participants.

### Variables

The selection of variables was guided by the recommendations of the Clinical Protocol and Therapeutic Guidelines of the Ministry of Health, which direct the monitoring and management of the condition within the scope of PC^2^. Variables were analyzed based on corresponding questions from the 2019 PNS.

The outcome variable was defined based on the question, “*Is your home registered with the family health unit?*” and was dichotomized as 1 (Yes) and 0 (No). Responses of “Don’t know” were excluded from the analysis and treated as missing data. Explanatory variables were selected to represent indicators of care and access to health services among adults diagnosed with DM, as detailed below:

• Last blood glucose check, assessed by the question, “*When was the last time you had a blood test to measure your blood glucose?*” Responses were recoded into two categories: “less than 6 months” and “6 months or more”;
• Use of medication for diabetes management, a combined variable was created based on two questions from the 2019 PNS questionnaire: “*In the past two weeks, have you used insulin to control diabetes?*” and “*In the past two weeks, have you used any medication to control diabetes?*” Individuals who answered “yes” to at least one of these questions were classified as “yes” for the use of insulin and/or oral medications;
• Acquisition of at least one diabetes medication through the “*Aqui Tem Farmácia Popular*” program, based on the following questions: “*Was any oral diabetes medication obtained through ‘Aqui Tem Farmácia Popular’?*” and “*Was insulin obtained through ‘Aqui Tem Farmácia Popular’?*” Responses were categorized as “yes” or “no,” with individuals classified as “yes” if they reported obtaining at least one of the medications through this program;
• Receipt of medical care for diabetes, based on the question “*When was the last time you received medical care for diabetes?*” Responses were recoded as “yes” if care had occurred within the past 12 months, and “no” otherwise;
• Location of the last medical consultation, based on the question: “*During your last medical visit for diabetes, where were you attended?*” Responses were categorized as “yes” if care occurred at a Primary Health Care Unit (*Unidade Básica de Saúde* - UBS), and “no” for all other options;
• Care by the same health professional in previous consultations, based on the question “*During your last consultation, was the physician the same as in previous consultations?*” with response options “yes” and “no;”
• Hospitalization due to diabetes or related complications, based on the question, “*Have you ever been hospitalized due to diabetes or any related complication?*” (“yes” or “no”);
• Eye examination in the past year, based on the question “*When was the last time you had an eye exam or fundoscopy in which your pupil was dilated?*” For this variable, the response “never had” was recoded as “no,” while all other responses were classified as “yes;”
• Foot examination in the past year, based on the question “*When was the last time a physician or health professional examined your feet to check for sensation or the presence of wounds or irritations?*” The response “never had feet examined” was categorized as “no,” and all other responses as “yes;”
• Degree of limitation in usual activities, based on the question: “*In general, to what extent does diabetes or any diabetes-related complication limit your usual activities?*” Responses “does not limit,” “a little,” and “moderately” were recoded as “limits a little;” while “intensely” and “very intensely” were grouped as “limits intensely;”
• Consultations with specialist physicians, assessed by the question: “*In any of your diabetes-related appointments, were you referred to a specialist physician, such as a cardiologist, endocrinologist, nephrologist, or ophthalmologist?*” The response “yes” was retained as the positive category, while all other responses were recoded as “no.”

### Statistical analysis

Prevalence rates and their corresponding 95% confidence intervals (95% CI) for care indicators and access to primary care services among the Brazilian population with DM were calculated according to the variables described.

Subsequently, a bivariate analysis was conducted to examine the association between household registration at a Family Health Unit (*Unidade de Saúde da Família* - USF) and the selected indicators. Proportions and 95%CI were used for this analysis. Associations between variables were evaluated using Pearson’s χ² test, with p≤0.05 considered statistically significant. Indicators showing a significant association (p≤0.05) in the bivariate analysis were then included in a logistic regression analysis (model 1) to estimate the odds ratio (OR). These indicators were subsequently incorporated into a second model (model 2) to estimate ORs adjusted for gender and age.

All analyses accounted for the sampling design and sample weights to ensure representative population estimates. Analyses were performed using the *svy* module of Stata Statistical Software, version 16.

### Ethical aspects

This study was based on secondary data and, therefore, did not require submission to a Research Ethics Committee. The original PNS project was previously approved by the National Research Ethics Committee (*Comitê Nacional de Ética em Pesquisa* - Conep), under opinion No. 3.529.376^12^ ^,^ ^13^.

## Data Availability Statement:

The complete dataset supporting the findings of this study is publicly available through SciELO Data and can be accessed at https://www.ibge.gov.br/estatisticas/sociais/saude/9160-pesquisa-nacional-de-saude.html.

## RESULTS

Among the Brazilian adult population, 8.2% (95%CI 7.9-8.6) self-reported a diagnosis of DM. Of these individuals, 75.4% (95%CI 73.5-77.2) reported that their last blood glucose measurement occurred more than six months ago, and 96% (95%CI 95.1-96.8) reported using medication or insulin in the previous two weeks. Additionally, 85.6% (95%CI 84.1-87.1) reported obtaining at least one medication through the “*Aqui Tem Farmácia Popular*” program, while 78.9% (95%CI 77.0-80.6) had received medical care for diabetes within the past year. Furthermore, 49.2% (95%CI 47.0-51.4) did not have their most recent consultation at an UBS, 59.2% (95%CI 56.9-61.4) were seen by the same doctor as in previous consultations, and 48.7% (95%CI 45.5-52.0) reported that all consultations after referral were conducted by a specialist. Regarding diabetes-related complications, 14.5% (95%CI 13.2-16.0) reported hospitalization, and 5.8% (95%CI 4.8-7.1) reported severe limitations in their usual activities. With respect to preventive examinations, 25.9% (95%CI 24-28) reported having an eye exam, and 35.7% (95%CI 33.6-37.8) reported having a foot exam within the past year (Table 1).

**Table 1.** Prevalence of diabetes mellitus and proportions of care and health service access indicators with 95% confidence intervals among Brazilian adults, PNS 2019.

Indicator	% (95%CI)
Diabetes mellitus	8.2 (7.9-8.6)
Last blood glucose measurement	
<6 months	24.5 (22.8-26.5)
≥6 months	75.4 (73.5-77.2)
Use of medication or insulin in the past two weeks	
No	3.9 (3.2-4.9)
Yes	96 (95.1-96.8)
At least one diabetes medication through “*Aqui Tem Farmácia Popular*”	
No	14.4 (12.9-15.9)
Yes	85.6 (84.1-87.1)
Received medical care for diabetes within the past year	
No	21.1 (19.3-23)
Yes	78.9 (77-80.6)
Last consultation at an UBS	
No	50.8 (48.6-52.9)
Yes	49.2 (47-51.4)
Attended by the same physician as previous consultations	
No	40.8 (38.6-43)
Yes	59.2 (56.9-61.4)
All consultations with a specialist physician after referral	
No	51.2 (48-54.5)
Yes	48.7 (45.5-52)
Hospitalization due to diabetes or complications	
No	85.5 (84-86.8)
Yes	14.5 (13.2-16)
Severe or very severe limitations in usual activities due to diabetes	
Limits a little	94.2 (92.9-95.2)
Limits intensely	5.8 (4.8-7.1)
Eye examination in the past year	
No	74 (72-76)
Yes	25.9 (24-28)
Foot examination in the past year	
No	64.3 (62.2-66.4)
Yes	35.7 (33.6-37.8)
N = 7,374	

The prevalence of adults diagnosed with DM in Brazil residing in households registered with an USF was 8.7% (95%CI 8.3-9.1). Among these individuals, 75% (95%CI 72.6-77.3) reported having measured their blood glucose within the past six months. The majority (96%; 95%CI 94.8-96.9) reported using medication or insulin in the previous two weeks. Approximately 86% (95%CI 84.3-87.3) indicated that they had obtained at least one medication for diabetes management through the “*Aqui Tem Farmácia Popular*” program (Table 2).

**Table 2.** Proportions and 95% confidence intervals of care and health access indicators among adults with diabetes mellitus, according to household registration in a Family Health Unit (USF) and estimates from unadjusted and adjusted models, PNS 2019.

Indicators	Household registered in USF			Model 1		Model 2	
	No	Yes	p-value	***Odds Ratio* % (95%CI)**	p-value	***Odds Ratio* % (IC95%)**	Valor p
Prevalence of diabetes mellitus	7.8 (7.2-8.5)	8.7 (8.3-9.1)					
Care indicators: proportion							
Last blood glucose measurement			0.4367				
<6 months	76.7 (72.9-80.1)	75 (72.6-77.3)					
Use of medication or insulin in the past two weeks			0.7037				
Yes	96.4 (94.1-97.8)	96 (94.8-96.9)					
At least one diabetes medication through “*Aqui Tem Farmácia Popular*”			0.5595				
Yes	85.1 (81.8-87.9)	86.1 (84.3-87.3)					
Received medical care for diabetes within the past year			0.7417				
Yes	78.5 (75.2-81.5)	79.2 (76.7-81.5)					
Last consultation at an UBS			<0.0001		<0.0001		<0.0001
Yes	36.8 (32.5-41.4)	55.5 (52.9-58)		2.14 (1.72-2.66)		2.13 (1.71-2.64)	
Attended by the same physician as previous consultations			0.0001		<0.0001		<0.0001
Yes	66.8 (62.4-70.8)	56.7 (54.1-59.4)		0.65 (0.52-0.81)		0.65 (0.53-0.81)	
All consultations with a specialist physician after referral			<0.0001		<0.0001		<0.0001
Yes	39.8 (34.4-45.4)	56.9 (52.9-60.8)		2 (1.51-2.65)		1.97 (1.49-2.60)	
Hospitalization due to diabetes or complications			0.0139				
Yes	11.5 (9.3-14.2)	15.6 (13.9-17.6)					
Severe or very severe limitations in usual activities due to diabetes			0.0293		0.031		0.031
Limits intensely	4.1 (2.9-5.9)	6.9 (5.2-8.5)		1.65 (1.05-2.60)		1.65 (1.05-2.62)	
Eye examination in the past year			0.0043		0.004		0.005
Yes	30.1 (27.2-35)	24.5 (22.1-27)		0.72 (0.56-0.91)		0.73 (0.58-0.90)	
Foot examination in the past year			0.5652				
Yes	36.6 (32.6-40.8)	35.2 (32.7-37.8)					

Regarding medical care, 79.2% (95%CI 76.7-81.5) reported receiving care for diabetes within the past year; 55.5% (95%CI 52.9-58.0) had their most recent consultation at an UBS; 56.7% (95%CI 54.1-59.4) were seen by the same physician as in previous consultations; and 56.9% (95%CI 52.9-60.8) reported that all consultations following referral were conducted by a specialist. Concerning diabetes-related complications and limitations, 15.6% (95%CI 13.9-17.6) had been hospitalized due to complications, and 6.9% (95%CI 5.2-8.5) reported mild limitations in their usual activities. Finally, 24.5% (95%CI 22.1-27.0) reported having an eye exam, and 35.2% (95%CI 32.7-37.8) reported having a foot exam within the past year (Table 2).

Table 2 also presents estimates from the unadjusted model, which indicate that individuals with DM and a registered address at an USF had higher odds of having their most recent consultation at an UBS (OR=2.14; 95%I 1.72-2.66) and of being seen by a specialist following referral (OR=2; 95%CI 1.51-2.65). Conversely, these individuals had lower odds of being seen by the same physician as in previous consultations (OR=0.65; 95%CI 0.52-0.81) and of having undergone an eye exam within the past year (OR=0.72; 95%CI 0.56-0.91).

After adjustment, individuals with DM and a registered address at an USF continued to have higher odds of having their most recent consultation at an UBS (OR=2.13; 95%CI 1.71-2.64), of being seen by a specialist following referral (OR=1.97; 95%CI 1.49-2.60), and of reporting severe limitations in their usual activities due to the condition (OR=1.65; 95%CI 1.05-2.62). Conversely, they had lower odds of being seen by the same physician as in previous consultations (OR=0.65; 95%CI 0.53-0.81) and of having undergone an eye exam within the past year (OR=0.73; 95%CI 0.58-0.90). All associations remained statistically significant after adjustment (p<0.05) (Table 2).

## DISCUSSION

This study evaluated the care pathway for adults with DM in Brazil, based on the country’s most comprehensive household survey conducted in 2019. Among individuals with DM, 8.7% resided in households registered with an USF. In this group, there was a higher likelihood of having had the most recent consultation at an UBS and of being referred to specialist physicians compared with individuals from unregistered households. However, these individuals also reported a greater likelihood of experiencing significant limitations in daily activities due to the condition. Conversely, indicators of continuity of care were lower, as reflected by a smaller proportion of visits with the same healthcare professional and fewer eye examinations.

Primary Health Care Units, as components of the Health Care Network (*Rede de Atenção à Saúde* - RAS), represent the main entry point to the health system and are expected to facilitate access to comprehensive, effective, and continuous care, particularly in the management of NCDs^14^. A study conducted in Recife demonstrated that the “access” attribute of UBS received the highest score among evaluated attributes, primarily due to proximity to users’ residences^15^. In this context, household registration at an USF, combined with the principle of territorialization, is likely to strengthen the bond between users and healthcare teams, thereby promoting both access and continuity of clinical follow-up for NCDs such as diabetes^15^.

However, the high proportion of individuals not seen by the same physician in previous consultations underscores weaknesses in the continuity of care, a fundamental pillar of primary health care (PHC) and a key guideline of the Brazilian Unified Health System (*Sistema Único de Saúde* - SUS)^14^. Evidence suggests that continuity of care is associated with improved health outcomes, including reduced mortality, lower costs, and decreased healthcare utilization^16^ ^,^ ^17^. Discontinuity, in contrast, can result in lost referrals, fragmented care, and an increased risk of iatrogenic events, particularly for chronic conditions requiring ongoing follow-up, such as diabetes^14^. One study reported that individuals with diabetes mellitus who maintained continuity of care at the same healthcare unit or with the same provider were more likely to achieve adequate glycemic control compared with those without a fixed location or reference professional^18^. These findings underscore the importance of UBS as regular points of care and highlight the critical role of professional continuity in the effective management of diabetes.

More than half of individuals with diabetes residing in households registered with USF reported being referred to and treated by specialist physicians. This observation may reflect both the effectiveness of a well-coordinated RAS and potential limitations in the capacity of PHC to fully manage cases, a core guideline of its operation^18^. Primary health care should be capable of resolving most health problems within the population, including the management of NCDs^14^. Although referrals are integral to comprehensive care, their high frequency may indicate challenges faced by PHC teams in managing diabetes at the primary level, whether due to limited resources, insufficient training, or workload pressures. Evidence suggests that communication failures between different levels of care can lead to duplication of tests, therapeutic conflicts, and poorer patient experiences^19^. Further studies are warranted to determine whether referrals are made according to appropriate clinical criteria or whether they reflect structural and operational deficiencies in the routine functioning of PHC.

Diabetic retinopathy is a common complication of DM and can progress to irreversible vision loss if not detected early^20^. Performing regular eye examinations enables the early identification of retinal changes, allowing for timely intervention^20^ ^,^ ^21^. For individuals with DM1, an ophthalmological exam is recommended within five years of diagnosis, whereas for those with DM2, the exam should be performed immediately upon diagnosis^20^. Follow-up examinations should occur annually or at shorter intervals depending on the stage of retinopathy^20^. Pregnant women with diabetes should undergo eye examinations each trimester^20^. These considerations underscore the need to strengthen timely referrals, follow-up appointments, and health education initiatives that promote adherence to ophthalmological monitoring, particularly as only one-quarter of the studied population reported having an eye exam in the past year.

Finally, according to Andersen’s Behavioral Model, which posits that the use of health services is influenced by predisposing factors, enabling factors, and, primarily, by the perceived or evaluated need for care, individuals with greater functional impairment tend to have higher healthcare needs^22^. Consistent with the findings of this study, individuals with diabetes and a registered residence in an USF were more likely to report significant limitations in their usual activities due to comorbidities, reflecting this dynamic. This observation does not necessarily indicate inefficiency of the Family Health Strategy but may instead reflect greater utilization of services by individuals with higher clinical vulnerability, who are consequently more likely to be registered in an USF.

The limitations observed in the use of health services among certain population groups reflect socioeconomic inequalities that often extend beyond the governance of the health sector^23^. Unequal access to services among individuals with NCDs is strongly associated with factors such as education, health insurance coverage, and socioeconomic status, underscoring the need for intersectoral public policies to reduce social inequities^23^.

The limitations of this study are largely inherent to cross-sectional designs, in which exposure and outcome are assessed simultaneously, making it impossible to determine whether the exposure precedes or results from the outcome. Additionally, the use of self-reported data is subject to information bias, particularly recall bias. Despite these limitations, the findings provide a robust representation of Brazilian adults with DM, given the nationally representative sample and the use of weighted and stratified data adjustments.

These findings underscore disparities in the quality of care and access to PHC services among adults with DM in Brazil. Household registration at a Family Health Unit was associated with greater utilization of UBS services, more referrals to specialist physicians, and higher self-reported severe functional limitations. Conversely, lower continuity of care and fewer ophthalmological examinations were observed among registered individuals. Notably, the proportion of both ophthalmological and foot examinations was low in both groups, highlighting persistent weaknesses in the provision of preventive care.

Severe functional limitations were more frequent among low-income individuals registered in USF, suggesting that socioeconomic factors further negatively influence the healthcare of this vulnerable population. These findings emphasize the need to enhance continuity of care and the problem-solving capacity of primary health care, particularly in the management of chronic conditions such as DM.
