# Supplementary material for: Care and primary health care access indicators among Brazilian adults with diabetes mellitus, National Health Survey 2019
Source: Rev Bras Epidemiol. 2026 Mar 16;29:e260005. doi: 10.1590/1980-549720260005 (PMC12995340; doi:10.1590/1980-549720260005)
Supplement: Translation

# Translation: Indicadores de cuidado e acesso à atenção primária à saúde entre adultos brasileiros com diabetes mellitus, Pesquisa Nacional de Saúde, 2019

**DOI:** 10.1590/1980-549720260005.2

**Authors:** Luís Antônio Batista Tonaco, Bárbara Aguiar Carrato, Filipe Malta dos Santos, Regina Tomie Ivata Bernal, Deborah Carvalho Malta

**Affiliations:** I Universidade Federal de Minas Gerais, Escola de Enfermagem - Belo Horizonte (MG), Brasil.; II Fundação Educacional Lucas Machado - Belo Horizonte (MG), Brasil.

**Keywords:** Diabetes mellitus, Indicadores básicos de saúde, Atenção primária à saúde, Inquéritos epidemiológicos, Brasil

## Abstract

**Objetivo:**

Analisar os indicadores de acesso aos serviços de saúde e cuidado entre adultos com diabetes mellitus no Brasil, segundo a cobertura da atenção básica.

**Métodos:**

Estudo transversal, com dados da Pesquisa Nacional de Saúde (PNS) 2019. Foram selecionados apenas os indivíduos que autorreferiram diagnóstico de diabetes mellitus. As variáveis explicativas representaram indicadores de cuidado e acesso entre adultos com diagnóstico de diabetes mellitus. Foram calculados as prevalências e o Intervalo de Confiança de 95% (IC95%) dos indicadores. A associação com o cadastro em Unidade de Saúde da Família (USF) foi verificada por análise bivariada. Indicadores com p≤0,05 foram incluídos em modelo de regressão logística e, posteriormente, em modelo ajustado por sexo e idade.

**Resultados:**

Entre a população adulta brasileira, 8,2% (IC95% 7,9-8,6) autorrelataram diagnóstico de diabetes mellitus. Após ajuste, os indivíduos com diabetes e domicílio cadastrado em USF apresentaram maior chance de terem realizado a última consulta em uma Unidade Básica de Saúde (UBS) (OR=2,13; IC95% 1,71-2,64), de terem sido atendidos por médico especialista após encaminhamento (OR=1,97; IC95% 1,49-2,60) e de referir limitação intensa nas atividades habituais devido à condição (OR=1,65; IC95% 1,05-2,62). Por outro lado, observou-se menores chances de terem sido atendidos pelo mesmo médico das consultas anteriores (OR=0,65; IC95% 0,53-0,81) e de realizarem exame de vista no último ano (OR=0,73; IC95% 0,58-0,90).

**Conclusão:**

Existem disparidades no cuidado e no acesso aos serviços de atenção básica entre adultos brasileiros com diabetes.

## INTRODUÇÃO

O Diabetes Mellitus (DM) é uma doença crônica grave, caracterizada por concentrações elevadas de glicose no sangue, relacionadas aos efeitos da biologia anormal das células β sobre a ação da insulina^1^. A patologia é classificada em diferentes tipos, sendo o tipo 1 e o tipo 2 os mais prevalentes na população^2^. Várias complicações estão associadas ao DM, incluindo retinopatia, nefropatia, amputações, pé diabético, maior predisposição a doenças cardiovasculares e acidente vascular cerebral^2^ ^,^ ^3^. Dessa forma, o DM, quando não controlado, contribui para desfechos clínicos adversos, como hospitalizações frequentes, incapacidades funcionais e aumento da mortalidade^3^.

A condição afeta aproximadamente 3% da população mundial e apresenta tendência de crescimento até 2030^3^ ^,^ ^4^. A doença ocupa a 9ª posição entre as principais causas de Perda de Anos de Vida Ajustados por Incapacidade (DALYs) no mundo^4^. No contexto brasileiro, o diabetes mellitus também representa um expressivo problema de saúde pública^3^ ^,^ ^4^. Segundo dados da Pesquisa Nacional de Saúde (PNS) de 2013, a prevalência autorreferida da doença foi de 6,2%^3^. Em 2019, esta aumentou para 8%, com proporções significativamente mais altas entre indivíduos de baixos níveis socioeconômicos^3^.

Em 2014 o Ministério da Saúde instituiu uma portaria que estabelece parâmetros para estruturar as linhas de cuidados voltadas às pessoas com Doenças Crônicas Não Transmissíveis (DCNTs), incluindo o diabetes mellitus^5^. Essa iniciativa tem por objetivo aprimorar a atenção à saúde de pessoas com DCNTs, por meio de um acompanhamento contínuo e qualificado^5^ ^,^ ^6^. Com isso, busca-se prevenir complicações, reduzindo hospitalizações e óbitos evitáveis^5^ ^,^ ^6^.

O acesso aos serviços de saúde constitui um elemento essencial para modificar de forma positiva o curso das doenças e promover a saúde^7^. De modo geral, esse conceito refere-se à utilização oportuna e adequada dos serviços disponíveis, com vistas à obtenção dos melhores desfechos em saúde^7^. No Brasil, o acesso aos serviços de saúde é promovido por meio de programas como a Estratégia Saúde da Família (ESF), que fortalecem a capilaridade da Atenção Básica (AB) no território nacional, promovendo ações de prevenção, diagnóstico precoce e acompanhamento contínuo^8^ ^,^ ^9^.

No contexto do diabetes, a AB deve assegurar não apenas o acesso oportuno, mas também o seguimento clínico contínuo, a realização regular de exames e ações educativas voltadas ao autocuidado^10^. Nesse sentido, acompanhar os indicadores relacionados ao manejo e à assistência em saúde é importante para avaliar a qualidade do cuidado oferecido às pessoas com DM, uma vez que possibilita identificar desigualdades no acesso e na oferta de serviços, servindo como base para a formulação e o aprimoramento de políticas públicas.

A PNS representa uma importante ferramenta para a avaliação de tais indicadores, ao incluir perguntas sobre diagnóstico autorreferido de DM e os cuidados recebidos pela população acometida em seu questionário^6^. Assim, este estudo teve como objetivo analisar os indicadores de acesso aos serviços de saúde e cuidado entre adultos com diabetes mellitus no Brasil, segundo a cobertura da atenção básica.

## MÉTODOS

### Desenho do estudo e fonte de dados

Trata-se de um estudo transversal, com dados da PNS, edição 2019, realizada pelo Ministério da Saúde em parceria com o Instituto Brasileiro de Geografia e Estatística (IBGE). Os dados da PNS são de domínio público, disponíveis no repositório oficial da pesquisa^11^.

### Contexto

A PNS é um inquérito domiciliar de abrangência nacional, cujo questionário é estruturado em três partes: a primeira aborda as características do domicílio; a segunda é destinada a todos os moradores do domicílio; e a terceira é aplicada a um único morador selecionado, com 15 anos de idade ou mais^12^. Essa pesquisa possibilitou a obtenção de estimativas tanto para áreas urbanas quanto rurais, bem como para as macrorregiões do país, unidades federativas, capitais e regiões metropolitanas^12^.

### Participantes

A amostra final da PNS 2019 foi de 94.114 domicílios com entrevista realizada, com taxa de resposta de 93,6%^11^ ^,^ ^12^. A população-alvo foi composta por indivíduos com 15 anos ou mais, residentes em domicílios particulares permanentes, ou seja, construções destinadas exclusivamente à moradia^12^. Foram excluídos da amostra os domicílios situados em setores censitários com características especiais ou de baixa densidade populacional, como comunidades indígenas, instituições militares (quartéis e bases), alojamentos temporários, entre outros^12^. Informações detalhadas sobre a metodologia da pesquisa podem ser encontradas em publicações específicas^11^ ^,^ ^12^. Para este estudo, foram considerados apenas os indivíduos com 18 anos ou mais que responderam positivamente à pergunta: “*Algum médico já lhe deu o diagnóstico de diabetes?*”, totalizando uma população de 7.374 participantes.

### Variáveis

A seleção das variáveis baseou-se nas recomendações do Protocolo Clínico e Diretrizes Terapêuticas do Ministério da Saúde, que orienta as ações de acompanhamento e manejo da condição no âmbito da AB^2^. As variáveis foram analisadas com base em perguntas do questionário da PNS 2019.

O desfecho analisado foi construído de acordo com a questão “*O seu domicílio está cadastrado na unidade de saúde da família?*”, sendo dicotomizado em 1 (Sim) e 0 (Não). As respostas “Não sabe” foram desconsideradas da análise e tratadas como dados ausentes. As variáveis explicativas foram selecionadas com o propósito de representar indicadores de cuidado e de acesso aos serviços de saúde entre adultos com diagnóstico de diabetes mellitus, conforme descrito a seguir:

• Última verificação da glicemia, por meio da pergunta “*Quando foi a última vez que o(a) Sr.(a) fez exame de sangue para medir a glicemia?*”, sendo as respostas recodificadas em duas categorias: “menos de 6 meses” e “6 meses ou mais”;
• Para uso de medicamentos para o controle do diabetes, foi criada uma variável combinada com base em duas perguntas do questionário da PNS 2019: “*Nas duas últimas semanas, usou insulina para controlar o diabetes?*” e “*Nas duas últimas semanas, usou algum medicamento para controlar o diabetes?*”. Os indivíduos que responderam “sim” a pelo menos uma das perguntas foram classificados como “sim” ao uso de insulina e/ou medicamentos orais;
• Obtenção de pelo menos um medicamento para diabetes por meio do programa “Aqui Tem Farmácia Popular”, fundamentado nas seguintes perguntas: “*Algum dos medicamentos orais para diabetes foi obtido no ‘Aqui Tem Farmácia Popular’?*” e “*A insulina foi obtida no ‘Aqui Tem Farmácia Popular’?*”. As respostas foram categorizadas como “sim” ou “não”, considerando como “sim” os indivíduos que afirmaram ter obtido ao menos um dos medicamentos por esse meio;
• Recebimento de assistência médica para diabetes, com base na pergunta “*Quando foi a última vez que o(a) Sr.(a) recebeu atendimento médico por causa do diabetes?*”. As respostas foram recodificadas como “sim” quando o atendimento havia ocorrido há menos de 12 meses, e “não” nos demais casos;
• Local da última consulta médica, construída por meio da pergunta: “*Na última vez que recebeu atendimento médico para diabetes, onde o(a) Sr.(a) foi atendido?*”. As respostas foram categorizadas como “sim” quando o atendimento ocorreu em Unidade Básica de Saúde (UBS), e “não” para todas as demais opções;
• Atendimento com o mesmo profissional de saúde nas consultas anteriores, construída com base na pergunta “*Na última consulta, o médico que o(a) atendeu era o mesmo das consultas anteriores?*”, com opções de resposta “sim” e “não”;
• Internação por causa do diabetes ou de alguma complicação, baseada na questão “*Alguma vez o(a) Sr.(a) se internou por causa do diabetes ou de alguma complicação?*” (“sim” ou “não”);
• Realização de exame de vista no último ano, de acordo com a questão “*Quando foi a última vez que realizaram um exame de vista ou fundo de olho em que dilataram sua pupila?*”. Para essa variável, a resposta “nunca fez” foi recodificada como “não”, enquanto as demais foram classificadas como “sim”;
• Realização de exame dos pés no último ano, com base na pergunta “*Quando foi a última vez que um médico ou profissional de saúde examinou seus pés para verificar a sensibilidade ou presença de feridas ou irritações?*”. A resposta “nunca teve os pés examinados” foi categorizada como “não”, e as demais como “sim”;
• Grau de limitação nas atividades habituais, de acordo com a pergunta: “*Em geral, em que grau o diabetes ou alguma complicação do diabetes limita as suas atividades habituais?*”, sendo as respostas “não limita”, “um pouco” e “moderadamente” recodificadas como “limita pouco”; e as respostas “intensamente” e “muito intensamente” agrupadas como “limita intensamente”;
• Realização de consultas com médicos especialistas, verificadas pela pergunta: “*Em algum dos atendimentos para diabetes, houve encaminhamento para alguma consulta com médico especialista, tal como cardiologista, endocrinologista, nefrologista ou oftalmologista?*”. A resposta “sim” foi mantida como categoria positiva, enquanto todas as demais respostas foram recodificadas como “não”.

### Análises estatísticas

Foram calculados as prevalências e os respectivos Intervalos de Confiança de 95% (IC95%) dos indicadores de cuidado e acesso aos serviços de atenção básica da população brasileira com DM, conforme as variáveis mencionadas.

Em seguida, foi realizada uma análise bivariada com o objetivo de verificar a associação entre o cadastro do domicílio em Unidade de Saúde da Família (USF) e os indicadores selecionados. Para essa etapa, utilizou-se proporções e IC95%. A associação entre as variáveis foi testada por meio do teste do χ^2^ de Pearson, considerando-se p≤0,05 como nível de significância estatística. Em seguida, foi realizada análise de regressão logística para os indicadores que apresentaram associação estatisticamente significativa (p≤0,05) na análise bivariada, compondo o modelo 1 para estimar o *Odds Ratio* (OR). Posteriormente, esses indicadores foram incluídos em um segundo modelo (modelo 2), para estimar o OR ajustado por sexo e idade.

Todas as análises consideraram a estrutura amostral e os pesos amostrais para garantir estimativas representativas da população. As análises foram realizadas utilizando o módulo *svy* no *Stata Statistical Software*, versão 16.

### Aspectos éticos

Este estudo baseou-se na utilização de dados secundários e, portanto, dispensou a necessidade de submissão ao Comitê de Ética em Pesquisa. O projeto original da PNS foi previamente aprovado pelo Comitê Nacional de Ética em Pesquisa (Conep), sob o parecer n.º 3.529.376^12^ ^,^ ^13^.

## Declaração de disponibilidade de dados:

Todo o conjunto de dados que dá suporte aos resultados deste estudo foi disponibilizado no SciELO Data e pode ser acessado em https://www.ibge.gov.br/estatisticas/sociais/saude/9160-pesquisa-nacional-de-saude.html.

## RESULTADOS

Entre a população adulta brasileira, 8,2% (IC95% 7,9-8,6) autorrelataram diagnóstico de diabetes mellitus. Dos adultos brasileiros com a referida patologia, 75,4% (IC95% 73,5-77,2) referiram última mensuração de glicemia há mais de 6 meses, e 96% (IC95% 95,1-96,8) relataram uso de medicamento ou insulina nas duas últimas semanas. Além disso, 85,6% (IC95% 84,1-87,1) reportaram obter pelo menos um medicamento no programa “Aqui Tem Farmácia Popular”, enquanto 78,9% (IC95% 77,0-80,6) receberam assistência médica para diabetes há menos de um ano. Ainda, 49,2% (IC95% 47-51,4) não realizaram a última consulta em uma UBS, 59,2% (IC95% 56,9-61,4) foram atendidos pelo mesmo médico das consultas anteriores, e 48,7% (IC95% 45,5-52) tiveram todas as consultas conduzidas por médico especialista após encaminhamento. Quanto às complicações relacionadas ao diabetes, 14,5% (IC95% 13,2-16) relataram internação e 5,8% (IC95% 4,8-7,1), limitação intensa nas atividades habituais. Já no que tange aos exames preventivos, 25,9% (IC95% 24-28) referiram realização de exame de vista e 35,7% (IC95% 33,6-37,8), de exame dos pés no último ano (Tabela 1).

**Tabela 1.** Prevalência de diabetes mellitus e proporções dos indicadores de cuidado e acesso aos serviços de saúde com respectivos intervalos de confiança de 95% entre adultos brasileiros, PNS 2019.

Indicador	% (IC95%)
Diabetes mellitus	8,2 (7,9-8,6)
Última mensuração de glicemia	
<6 meses	24,5 (22,8-26,5)
≥6 meses	75,4 (73,5-77,2)
Uso de medicamento ou insulina nas duas últimas semanas	
Não	3,9 (3,2-4,9)
Sim	96 (95,1-96,8)
Pelo menos um medicamento para diabetes no programa “Aqui Tem Farmácia Popular”.	
Não	14,4 (12,9-15,9)
Sim	85,6 (84,1-87,1)
Recebeu assistência médica para diabetes há menos de um ano	
Não	21,1 (19,3-23)
Sim	78,9 (77-80,6)
Realizou a última consulta em uma UBS	
Não	50,8 (48,6-52,9)
Sim	49,2 (47-51,4)
Foi atendido pelo mesmo médico das consultas anteriores	
Não	40,8 (38,6-43)
Sim	59,2 (56,9-61,4)
Todas as consultas com médico especialista após encaminhamento	
Não	51,2 (48-54,5)
Sim	48,7 (45,5-52)
Internação por causa da diabetes ou alguma complicação	
Não	85,5 (84-86,8)
Sim	14,5 (13,2-16)
Grau intenso ou muito intenso de limitações nas atividades habituais devido a diabetes	
Limita pouco	94,2 (92,9-95,2)
Limita intensamente	5,8 (4,8-7,1)
Realização de exame de vista no último ano	
Não	74 (72-76)
Sim	25,9 (24-28)
Realização de exame dos pés no último ano	
Não	64,3 (62,2-66,4)
Sim	35,7 (33,6-37,8)
N = 7.374	

A prevalência de adultos com diagnóstico de diabetes mellitus no Brasil que residem em domicílios cadastrados em uma USF foi de 8,7% (IC95% 8,3-9,1). Dentre esses adultos, a proporção foi de 75% (IC95% 72,6-77,3) que referiram ter aferido a glicemia pela última vez há menos de 6 meses. A maioria (96%; IC95% 94,8-96,9) relatou ter feito uso de medicamento ou insulina nas duas últimas semanas. Cerca de 86% (IC95% 84,3-87,3) afirmaram ter obtido ao menos um medicamento para controle do diabetes por meio do programa “Aqui Tem Farmácia Popular” (Tabela 2).

**Tabela 2.** Proporções e respectivos intervalos de confiança de 95% dos indicadores de cuidado e acesso à saúde entre adultos com diabetes mellitus, segundo cadastro domiciliar em Unidade de Saúde da Família (USF) e estimativas dos modelos não ajustado e ajustado, PNS 2019.

Indicadores	Domicílio cadastrado na USF			Modelo 1		Modelo 2	
	Não	Sim	Valor p	***Odds Ratio* % (IC95%)**	Valor p	***Odds Ratio* % (IC95%)**	Valor p
Prevalência de diabetes mellitus	7,8 (7,2-8,5)	8,7 (8,3-9,1)					
Indicadores de cuidado: proporção							
Última mensuração de glicemia			0,4367				
<6 meses	76,7 (72,9-80,1)	75 (72,6-77,3)					
Uso de medicamento ou insulina nas duas últimas semanas			0,7037				
Sim	96,4 (94,1-97,8)	96 (94,8-96,9)					
Pelo menos um medicamento para diabetes no programa “Aqui Tem Farmácia Popular”			0,5595				
Sim	85,1 (81,8-87,9)	86,1 (84,3-87,3)					
Recebeu assistência médica para diabetes há menos de um ano			0,7417				
Sim	78,5 (75,2-81,5)	79,2 (76,7-81,5)					
Realizou a última consulta em uma UBS			<0,0001		<0,0001		<0,0001
Sim	36,8 (32,5-41,4)	55,5 (52,9-58)		2,14 (1,72-2,66)		2,13 (1,71-2,64)	
Foi atendido pelo mesmo médico das consultas anteriores			0,0001		<0,0001		<0,0001
Sim	66,8 (62,4-70,8)	56,7 (54,1-59,4)		0,65 (0,52-0,81)		0,65 (0,53-0,81)	
Todas as consultas com médico especialista após encaminhamento			<0,0001		<0,0001		<0,0001
Sim	39,8 (34,4-45,4)	56,9 (52,9-60,8)		2 (1,51-2,65)		1,97 (1,49-2,60)	
Internação por causa de diabetes ou alguma complicação			0,0139				
Sim	11,5 (9,3-14,2)	15,6 (13,9-17,6)					
Grau intenso ou muito intenso de limitações nas atividades habituais devido a diabetes			0,0293		0,031		0,031
Limita intensamente	4,1 (2,9-5,9)	6,9 (5,2-8,5)		1,65 (1,05-2,60)		1,65 (1,05-2,62)	
Realização de exame de vista no último ano			0,0043		0,004		0,005
Sim	30,1 (27,2-35)	24,5 (22,1-27)		0,72 (0,56-0,91)		0,73 (0,58-0,90)	
Realização de exame dos pés no último ano			0,5652				
Sim	36,6 (32,6-40,8)	35,2 (32,7-37,8)					

Quanto à assistência médica, 79,2% (IC95% 76,7-81,5) relataram ter recebido atendimento médico para diabetes há menos de um ano; 55,5% (IC95% 52,9-58), ter realizado a última consulta em uma UBS; 56,7% (IC95% 54,1-59,4), ter sido atendidos pelo mesmo médico das consultas anteriores; ainda, 56,9% (IC95% 52,9-60,8) tiveram todas as consultas conduzidas por um médico especialista após encaminhamento. No que tange às complicações e limitações decorrentes da diabetes, 15,6% (IC95% 13,9-17,6) foram internados devido a complicações, e 6,9% (IC95% 5,2-8,5) relataram pouca limitação nas atividades habituais. Por fim, 24,5% (IC95% 22,1-27) realizaram exame de vista no último ano e 35,2% (IC95% 32,7-37,8), exame dos pés no último ano (Tabela 2).

Na Tabela 2 são apresentadas, também, as estimativas do modelo não ajustado, nas quais se observa que indivíduos com DM e domicílio cadastrado em USF apresentaram maior chance de ter realizado a última consulta em uma UBS (OR=2,14; IC95% 1,72-2,66) e de terem sido atendidos por médico especialista após encaminhamento (OR=2; IC95% 1,51-2,65). Por outro lado, tiveram menor chance de ser atendidos pelo mesmo médico das consultas anteriores (OR=0,65; IC95% 0,52-0,81) e de realizarem exame de vista no último ano (OR=0,72; IC95% 0,56-0,91).

Após ajuste, os indivíduos com DM e domicílio cadastrado em USF continuaram apresentando maior chance de ter realizado a última consulta em uma UBS (OR=2,13; IC95% 1,71-2,64), de terem sido atendidos por médico especialista após encaminhamento (OR=1,97; IC95% 1,49-2,60) e de referir limitação intensa nas atividades habituais devido à condição (OR=1,65; IC95% 1,05-2,62). Por outro lado, observou-se menores chances de terem sido atendidos pelo mesmo médico das consultas anteriores (OR=0,65; IC95% 0,53-0,81) e de realizarem exame de vista no último ano (OR=0,73; IC95% 0,58-0,90). Todas essas associações foram estatisticamente significativas após o ajuste (p<0,05) (Tabela 2).

## DISCUSSÃO

O presente estudo avaliou a linha do cuidado em adultos com diabetes mellitus no Brasil, segundo o inquérito domiciliar mais importante do país realizado em 2019. Foram 8,7% os indivíduos com DM nos domicílios com cadastro em USF. Nesse grupo, observou-se maior chance de terem realizado a última consulta em uma UBS e sido encaminhados a médicos especialistas quando comparado ao grupo de domicílios não cadastrados. Contudo, também se observou maior chance de referirem limitação intensa em atividades habituais devido à condição quando comparado ao grupo de domicílios não cadastrados. Por outro lado, observou-se menor continuidade do cuidado, refletida na menor proporção de atendimento com o mesmo profissional de saúde e na realização de exame de vista.

As Unidades Básicas de Saúde, enquanto componentes da Rede de Atenção à Saúde (RAS), constituem a principal porta de entrada do sistema de saúde e devem atuar como facilitadoras do acesso a cuidados integrais, resolutivos e contínuos, principalmente no contexto de enfrentamento das DCNTs^14^. Estudo realizado em Recife demonstrou que o atributo “acesso” da UBS obteve o escore mais elevado entre os avaliados, impulsionado pela proximidade domiciliar^15^. Nesse sentido, o cadastro domiciliar em uma USF, aliado à lógica da territorialização, tende a fortalecer o vínculo entre usuário e equipe, favorecendo tanto o acesso quanto a continuidade do acompanhamento clínico de DCNTs, como o diabetes^15^.

No entanto, a elevada proporção de indivíduos que não foram atendidos pelo mesmo médico em consultas anteriores evidencia fragilidades na longitudinalidade do cuidado, um dos pilares da Atenção Primária à Saúde (APS) e uma diretriz do Sistema Único de Saúde (SUS)^14^. Evidências indicam que a continuidade do cuidado está associada a melhores desfechos em saúde, incluindo redução na mortalidade, nos custos e na utilização de cuidados de saúde^16^ ^,^ ^17^. Nesse contexto, sua descontinuidade pode levar à perda de referências, à fragmentação do cuidado e ao aumento do risco de iatrogenias, especialmente em condições crônicas que requerem acompanhamento, como o diabetes^14^. Estudo identificou que pessoas com DM que mantinham continuidade no local de atendimento ou no prestador de cuidados apresentaram maior probabilidade de alcançar controle glicêmico adequado, em comparação àquelas sem um local fixo ou profissional de referência^18^. Esse achado reforça a importância da UBS como ponto regular de cuidado e destaca o papel da continuidade profissional no manejo eficaz do diabetes.

Mais da metade dos indivíduos com diabetes e domicílios cadastrados em USF relataram ter sido encaminhados e atendidos por médicos especialistas. Esse achado pode indicar tanto um êxito de uma RAS bem articulada quanto possíveis limitações na resolutividade da APS, outra importante diretriz a ser por esta operacionalizada^18^. A Atenção Primária à Saúde deve ser capaz de resolver a maior parte dos problemas de saúde da população, incluindo o manejo de DCNTs^14^. Embora os encaminhamentos façam parte do cuidado integral, sua alta frequência pode indicar dificuldades das equipes em lidar com casos de diabetes no nível primário, seja por falta de recursos, capacitação ou sobrecarga. Estudo mostra que, quando há falhas na comunicação entre diferentes níveis de atenção, ocorre duplicação de exames, conflitos terapêuticos e pior experiência para o usuário^19^. Assim, estudos adicionais são necessários para avaliar se os encaminhamentos ocorrem por critérios clínicos adequados ou se revelam falhas estruturais e operacionais no cotidiano da APS.

Além disso, a retinopatia diabética é uma das complicações do DM e pode evoluir para perda visual irreversível se não for identificada precocemente^20^. Assim, a realização do exame de vista possibilita a detecção precoce de alterações retinianas, permitindo intervenções oportunas^20^ ^,^ ^21^. Para indivíduos com DM1, o exame oftalmológico deve ser feito até cinco anos após o diagnóstico; em casos de DM2, imediatamente após^20^. A reavaliação deve ser anual ou em intervalos menores, conforme o estágio da retinopatia^20^. Gestantes com diabetes, por sua vez, devem realizar o exame a cada trimestre^20^. Tal cenário reforça a necessidade de fortalecer encaminhamentos oportunos, retorno com contrarreferência e ações de educação em saúde que estimulem a adesão ao acompanhamento oftalmológico, à medida que apenas um quarto da população analisada realizou exame de vista no último ano.

Por fim, à luz do Modelo Comportamental de Andersen, segundo o qual o uso de serviços de saúde é determinado por fatores predisponentes, fatores facilitadores e, principalmente, pela necessidade percebida ou avaliada de cuidado, indivíduos com maior comprometimento funcional tendem a apresentar maior necessidade de atenção em saúde^22^. Nesse sentido, em concordância com os resultados deste estudo, usuários com diabetes e domicílio cadastrado em USF têm maior chance de referir limitação intensa em atividades habituais devido à comorbidade, que pode refletir essa dinâmica. Tal resultado não indica, necessariamente, ineficiência da Estratégia Saúde da Família, mas pode estar relacionado ao maior uso dos serviços por indivíduos em situação de maior vulnerabilidade clínica, os quais, por essa razão, têm mais chances de estarem cadastrados em USF.

Cabe destacar, ainda, que as limitações observadas na utilização dos serviços de saúde entre determinados grupos populacionais refletem desigualdades socioeconômicas que extrapolam, muitas vezes, a governabilidade do setor saúde^23^. Nesse contexto, o acesso desigual aos serviços entre indivíduos com DCNTs está fortemente associado a fatores como escolaridade, posse de plano de saúde e condição socioeconômica, indicando a necessidade de políticas públicas intersetoriais voltadas à redução das iniquidades sociais^23^.

As limitações deste estudo são, em grande parte, inerentes a delineamentos transversais, nos quais a exposição e o desfecho são determinados simultaneamente, o que impede de estabelecer se a exposição antecede ou é consequência do desfecho avaliado. Além disso, o uso de dados autorreferidos está sujeito a viés de informação, especialmente viés de memória. Apesar dessas limitações, os resultados deste estudo representam de forma significativa a realidade dos adultos brasileiros com diabetes mellitus, considerando que a amostra possui abrangência nacional e que os dados foram ajustados por pesos amostrais e estratificação.

Por fim, estes resultados evidenciam disparidades na qualidade do cuidado e no acesso aos serviços de AB entre adultos com DM no Brasil. Observou-se que o cadastro domiciliar em Unidade de Saúde da Família está associado a maior utilização da UBS como local de atendimento, maior encaminhamento a especialistas e maior autorrelato de limitações funcionais intensas. Por outro lado, identificou-se menor continuidade do cuidado e menor realização de exames oftalmológicos entre os cadastrados. Destaca-se, ainda, que a proporção de realização de exames oftalmológicos e dos pés foi baixa em ambos os grupos analisados, revelando fragilidades comuns na oferta de ações preventivas.

As limitações funcionais intensas foram mais frequentes entre indivíduos de baixa renda e cadastrados em USF, sugerindo que fatores socioeconômicos também contribuem de forma negativa para o cuidado em saúde dessa população em situação de maior vulnerabilidade. Tais achados ressaltam a necessidade de aprimorar a longitudinalidade do cuidado e a capacidade resolutiva da Atenção Primária à Saúde, especialmente no manejo de condições crônicas como o diabetes mellitus.
